# Tumor-Derived Exosomes Modulate Primary Site Tumor Metastasis

**DOI:** 10.3389/fcell.2022.752818

**Published:** 2022-03-02

**Authors:** Suwen Bai, Zunyun Wang, Minghua Wang, Junai Li, Yuan Wei, Ruihuan Xu, Juan Du

**Affiliations:** ^1^ Longgang District People´s Hospital of Shenzhen, The Second Affiliated Hospital of The Chinese University of Hong Kong, Shenzhen, China; ^2^ School of Basic Medical Sciences, Anhui Medical University, Hefei, China

**Keywords:** tumor-derived exosomes, metastasis, pre-metastatic niche, angiogenesis, immunosuppression

## Abstract

Tumor-derived exosomes (TDEs) are actively produced and released by tumor cells and carry messages from tumor cells to healthy cells or abnormal cells, and they participate in tumor metastasis. In this review, we explore the underlying mechanism of action of TDEs in tumor metastasis. TDEs transport tumor-derived proteins and non-coding RNA to tumor cells and promote migration. Transport to normal cells, such as vascular endothelial cells and immune cells, promotes angiogenesis, inhibits immune cell activation, and improves chances of tumor implantation. Thus, TDEs contribute to tumor metastasis. We summarize the function of TDEs and their components in tumor metastasis and illuminate shortcomings for advancing research on TDEs in tumor metastasis.

## Background

Exosomes are extracellular vesicles, approximately 30–150 nm in diameter, that contain functional biomolecules, such as proteins, RNA, DNA, and lipids, and can interact with recipient cells (Balaj et al., 2011; Choi et al., 2013; Peinado et al., 2011; Raposo and Stoorvogel, 2013; Skog et al., 2008; Thakur et al., 2014; Thery et al., 2009; Valadi et al., 2007). Exosomes are present in various body fluids and are regarded as a key component of intercellular communication. Tumor cell-, stromal cell-, or even normal cell–derived exosomes play an important role in tumor progression and can induce angiogenesis and accelerate metastasis (Hood et al., 2011; Luga et al., 2012; Peinado et al., 2012). The components and functions of the exosomes depend on the cell types; some studies have shown many differences in the contents and release rate in different types of cells. But, the complete mechanism and process have not yet been elucidated and need to be further explored. Metastasis is the leading cause of tumor-induced death and is a complex process involving local invasion, survival, and evasion from immunosurveillance, invasion into circulation, and extravasation at secondary organs (Fidler and Kripke, 2015; Wan et al., 2013). Tumor-derived exosomes (TDEs) are a significant component of the tumor microenvironment and are involved in promoting tumor metastasis through several mechanisms, including acquiring primary tumor migration capacity, tumor angiogenesis, escaping immune system organotropic metastasis, forming the pre-metastatic niche, and metastatic tumor growth in the secondary site.

**FIGURE 1:**
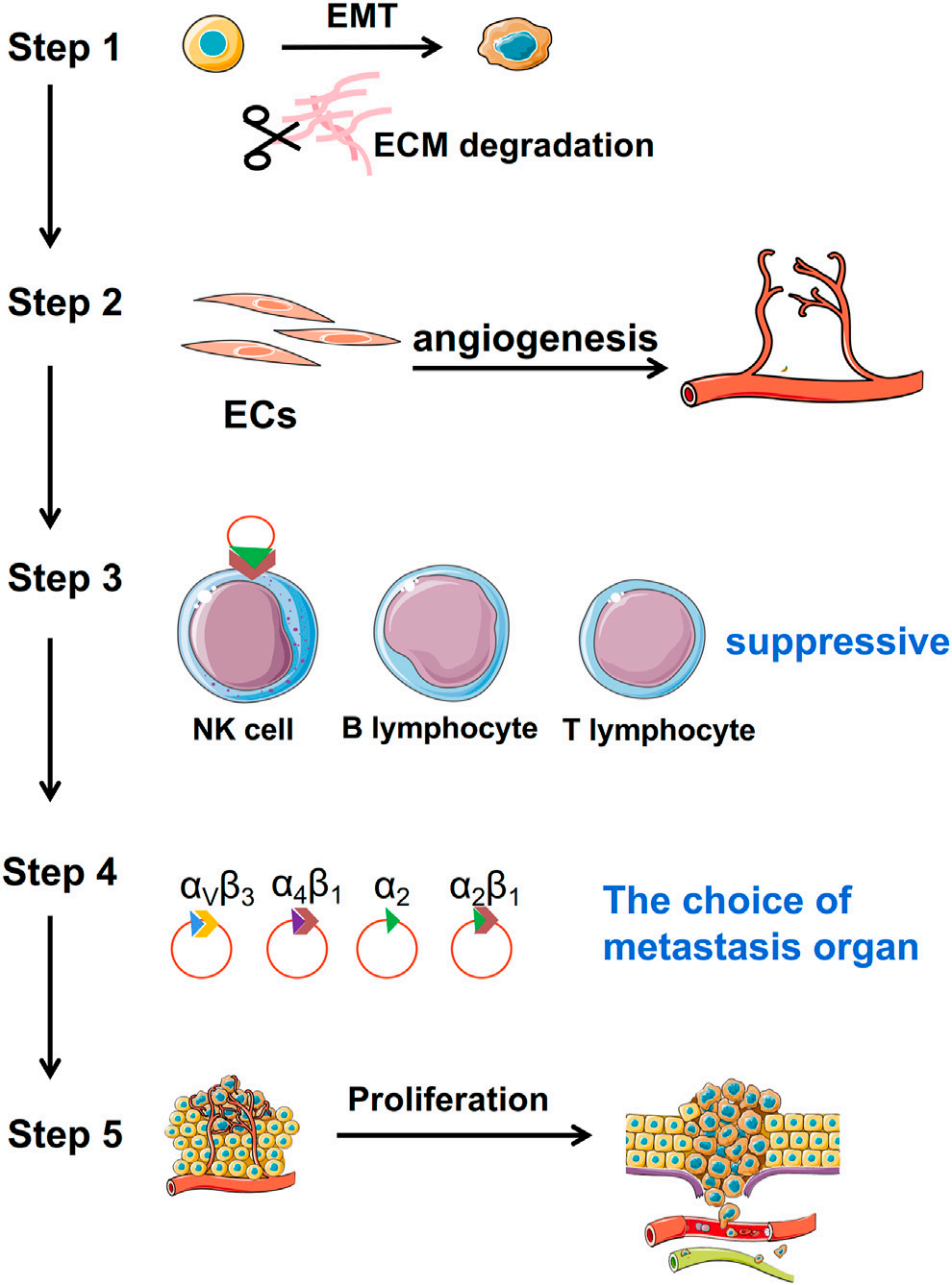
Function of TDEs in tumor metastasis. TDEs are mainly involved in tumor metastasis through five aspects. Step 1: acquisition of tumor migration ability; Step 2: angiogenesis; Step 3: immunosuppression; Step 4: localization of metastatic sites; and Step 5: enhancement of proliferation ability of tumor cells after migration.

In this review, we summarize the function of exosomes in every aspect of cancer metastasis (Figure 1) to provide a better systematic comprehension of the role of exosomes in tumor metastasis and propose practical implications of early diagnosis, treatment, and prognostic methods for cancer.

## Tumor-Derived Exosomes Enhance the Migration Ability of Tumor Cells

### Tumor-Derived Exosomes Promote Epithelial–Mesenchymal Transition to Initiate Metastasis

Epithelial–mesenchymal transition (EMT) frequently initiates the metastatic process (Li et al., 2021). Epithelial tumor cells acquire mesenchymal characteristics under the influence of cancer-associated fibroblasts (CAFs) in the tumor stroma (Diepenbruck and Christofori, 2016). Epithelial markers, including E-cadherin, zona occludens 1 (ZO-1), cytokeratins, desmoplakin, and laminin, are downregulated, and mesenchymal markers, including N-cadherin, β-catenin, and vimentin, are upregulated (Sommers et al., 1994; Li Y. et al., 2019). During EMT, tumor cells lose their apical–basal polarity, basement anchoring, and cell–cell junctions and switch to a low proliferation state with enhanced migratory and invasion capabilities (Basil et al., 2020). Once the tumor cells reach a distant pre-metastatic niche, the reversed process takes place (Maren, 2016). This so-called mesenchymal–epithelial transition (MET) returns tumor cells to a high proliferative state and enables the formation of micrometastases (Bakir et al., 2019). TDEs play an important regulatory role in mediating the EMT and MET transformation (Bigagli et al., 2019). There has been increasing research showing the signaling pathway involved in inducing cancer-related EMT. We propose that the critical components in TDEs can serve to promote EMT.

**FIGURE 2:**
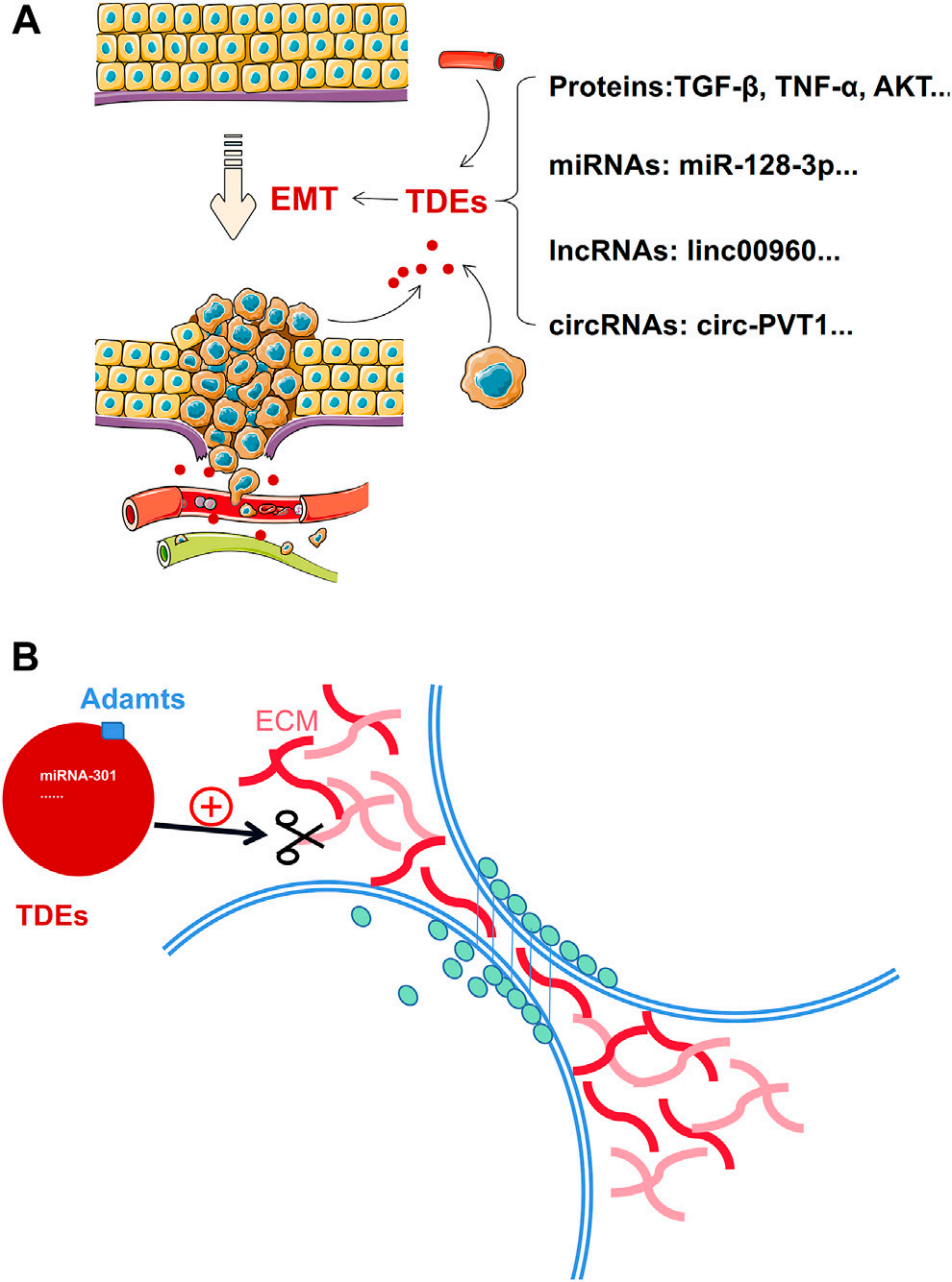
TDEs enhance the migration ability of tumor cells by promoting EMT and degrading the ECM. **(A):** Exosomes carry proteins, miRNA, lncRNA, and circRNA to promote the occurrence and development of EMT. **(B)**: TDEs carry proteins or non-coding RNA to initiate degradation of the ECM.

The latest hypothesis is TDEs may be conduits for initiating signals for EMT. For example, TDEs carry EMT derivers, such as transforming growth factor-beta (TGF-β), tumor necrosis factor-alpha (TNF-α), hypoxia-inducible factor 1 alpha (HIF-1α), protein kinase B (AKT), caveolin-1, platelet-derived growth factors (PDGFs), and β-catenin Wnt pathway modulators, that directly enhance the process of EMT (Aga et al., 2014; Kucharzewska et al., 2013; Luga et al., 2012; Ramteke et al., 2015). TDEs transmit non-coding RNA, such as, miR-128-3p, miR-27, LINC00960, linc02470, circ-PVT1, and circ-CPA4, that upregulate EMT (Huang C.-S. et al., 2020; Liu et al., 2019; Wang J. et al., 2018). Therefore, many studies have shown that tumor cells can secrete exosomes into the extracellular space and promote the EMT through their effectors: proteins, miRNAs, circRNAs, and lncRNAs (Figure 2A).

### Tumor-Derived Exosomes Promote Extracellular Matrix Degradation

The extracellular matrix (ECM) is a scaffold for tissues and organs (Eble and Niland, 2019). The ECM is a complex network combined with proteins, proteoglycans, and glycoproteins that can regulate cell growth, survival, motility, and differentiation through specific receptors, such as integrin, syndecan, and discoidin receptors (Leitinger, 2011; Xian et al., 2010). Cancer-associated ECM is not only an integral feature of a tumor but also actively contributes to its histopathology and malignant behavior (Levental et al., 2009; Provenzano et al., 2008). From tumor initiation to metastasis, ECM molecules bind with cell surface receptors and activate intracellular signaling pathways. ECM adhesion–induced signals promote self-sufficient growth through mitogen-activated protein kinase (MAPK) and phosphatidylinositol 3-kinase (PI3K) (Pylayeva et al., 2009). Focal adhesion kinase (FAK) signaling inhibits p15 and p21, which are growth suppressors, and p53 to limit the induction of apoptosis (Kim et al., 2008). TGF-β and RhoA/Rac signaling promote EMT induction and enhance promigratory pathways (Leight et al., 2012). The ECM can also enhance angiogenesis and strengthen vascular endothelial growth factor (VEGF) signaling in endothelial cells (Liu and Agarwal, 2010).

TDEs mediate tumor–tumor and tumor–host cell crosstalk (Kalluri, 2016). TDEs interact with and regulate the synthesis of ECM components and are involved in ECM remodeling (Rackov et al., 2018). The proteins on the surface of TDEs promote the activation of membrane-associated proteinases, such as Adamts1, Adamts4, and Adamts5, thus improving proteolytic activity (Ginestra et al., 1997; Lo Cicero et al., 2012). In addition, matrix metalloproteinases (MMPs) derived from TDEs participate in localized degradation and ECM proteolysis during cellular migration and metastasis (Ginestra et al., 1997; Atay et al., 2014). However, besides exosomal surface proteins, non-coding RNA also mediates ECM degradation. For example, breast cancer–derived exosomes carry miR-301 to regulate matrix modulation (Morad et al., 2020). Gastric cancer cell–derived exosomal miR-27a reshapes the ECM at adjacent sites and promotes tumor progression by downregulating CSRP2 expression and upregulating α-SMA expression (Wang et al., 2018). Currently, there are no direct reports on other non-coding RNAs, such as lncRNA and cicrRNA, but TDE-derived lncRNA and circRNA can influence fibroblast, chondrocyte, and epithelial cell function, secreting ECM components into the extracellular space (Tan et al., 2020).

Some data suggested that the ECM is a prerequisite for tumor cell invasion and metastasis (Tan et al., 2020). When the tumor cells metastasize, they detach from the ECM. Furthermore, the exosomes participate in this process (Figure 2B).

## Tumor-Derived Exosomes Promote Angiogenesis Directly or Indirectly

Regardless of tumor size, metastasis may occur; however, in most cases, metastasis is associated with large primary neoplasms (Fidler and Kripke, 2015). If a tumor mass exceeds 1 mm in diameter, angiogenesis is bound to occur (Folkman, 1971; Nagy and Dvorak, 2012). Therefore, exploring tumor angiogenesis is an important way to understand tumor metastasis.

### Tumor-Derived Exosomes Promote Angiogenesis by Activating Macrophages

Cancer-derived exosomes stimulate macrophage infiltration and polarization for establishing a pre-metastatic niche. For example, exosomes derived from CT-26, a colon cancer cell, can provoke macrophages to secrete significantly high levels of monocyte chemoattractant protein-1 (MCP-1) and TNF, thus promoting the growth and migration of colorectal cancer cells. Lung cancer cell–derived exosomes containing miRNA-103 upregulate angiogenic VEGF-A and angiopoietin-1 expression from M2 macrophages (Hsu et al., 2018; Wu et al., 2019). Therefore, TDEs can motivate the angiogenic property of macrophages such as secretion of VEGF (Wu et al., 2019). It can induce angiogenesis by tumor cells. In addition, other immune cells also contribute to tumor angiogenesis, such as neutrophils, myeloid precursor cells (MPCs), and dendritic cells (DCs) (Albini et al., 2018). But, there are no reports about TDE-educated neutrophils, MPCs, or DCs to promote angiogenesis in metastasis.

### Tumor-Derived Exosomes Carry Non-coding RNA and Proteins to Promote Angiogenesis Directly

TDEs carry non-coding RNAs, including microRNA, lncRNA, and circRNA, that play an indispensable role in angiogenesis. TDEs carry miR-25-3p that regulates VEGFR2, ZO-1, occludin, and claudin5 expression in ECs by targeting KLF2 and KLF4 and eventually promotes vascular permeability and angiogenesis (Felcht et al., 2012; Wu et al., 2019). TDEs deliver miR-130a to vascular cells to promote angiogenesis by targeting c-MYB (Yang et al., 2018). Exosomal miR-155-5p can induce angiogenesis through the SOCS1/JAK2/STAT3 signaling pathway (Zhou X. et al., 2018). Exosomal miR-135b promotes angiogenesis by inhibiting FOXO1 expression. Exosomal miR-23a induces angiogenesis by targeting TSGA10, prolyl hydroxylase, tight junction protein ZO-1, and SIRT1 (Sruthi et al., 2018; Bai et al., 2019). Exosomal miR-1229 promotes angiogenesis by targeting HIPK2. Exosomal miR-21 promotes angiogenesis by targeting STAT3 (Liu and Cao et al., 2016). In addition, lncRNA containing lncCCAT2, lncMALAT1, lncRNA-p21, and lncPOU3F3 or circRNA, such as TDE-derived circRNA-100338, also promote angiogenesis (Castellano et al., 2020; Huang X.-Y. et al., 2020; Lang, Hu, & Chen et al., 2017; Lang, Hu, &; Zhang Z. et al., 2017; Qiu J.-J. et al., 2018). LncRNA and circRNA are often used as “sponges” to regulate miRNA expression in cells. Moreover, TDEs carry a variety of angiogenic proteins, such as VEGF, IGFBP3, MMP2, ICAM-1, and IL-8, thus enhancing angiogenesis through *in vitro* and *in vivo* ligand/receptor signaling (Ludwig and Whiteside, 2018). Therefore, a combination of multiple non-coding RNAs and exosomal proteins promotes tumor angiogenesis.

**FIGURE 3:**
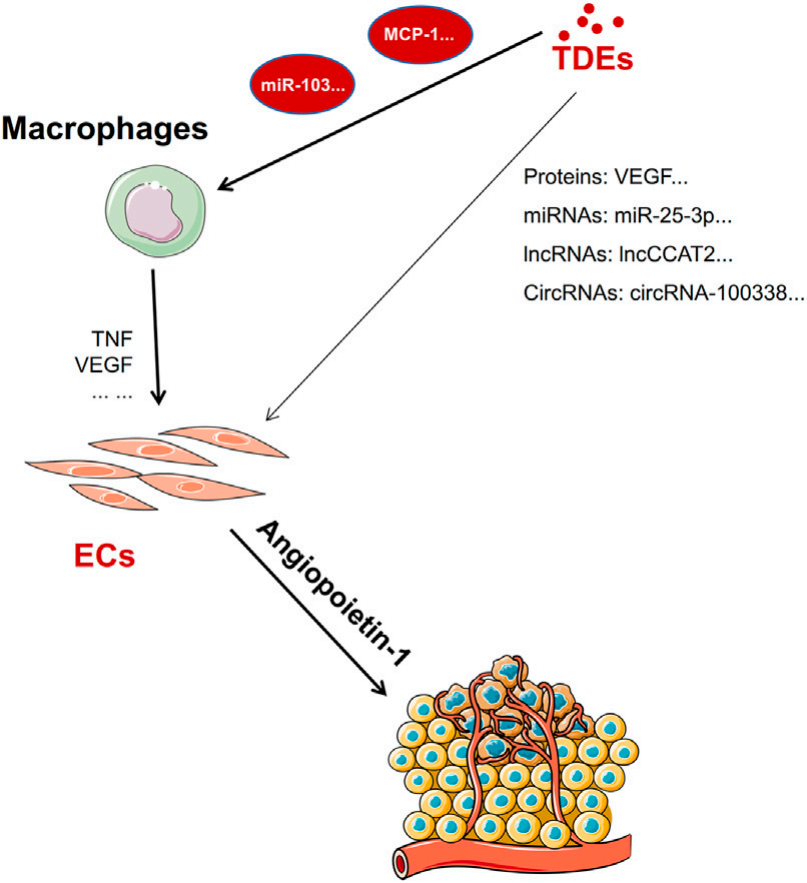
Exosomes directly or indirectly promote angiogenesis. TDEs promote macrophages to release TNF and VEGF to promote angiogenesis factors by carrying miRNAs or proteins. In addition, TDEs carry proteins, miRNAs, lncRNA, or circRNA to promote angiogenesis directly by targeting endothelial cells.

The importance of angiogenesis in tumor metastasis cannot be understated, TDEs can carry proteins and non-coding RNAs that directly promote angiogenesis or they can mediate angiogenesis indirectly by “educating” macrophages to release proangiogenic factors (Figure 3).

## Tumor-Derived Exosomes can Protect Tumor Cells During Metastasis

**FIGURE 4:**
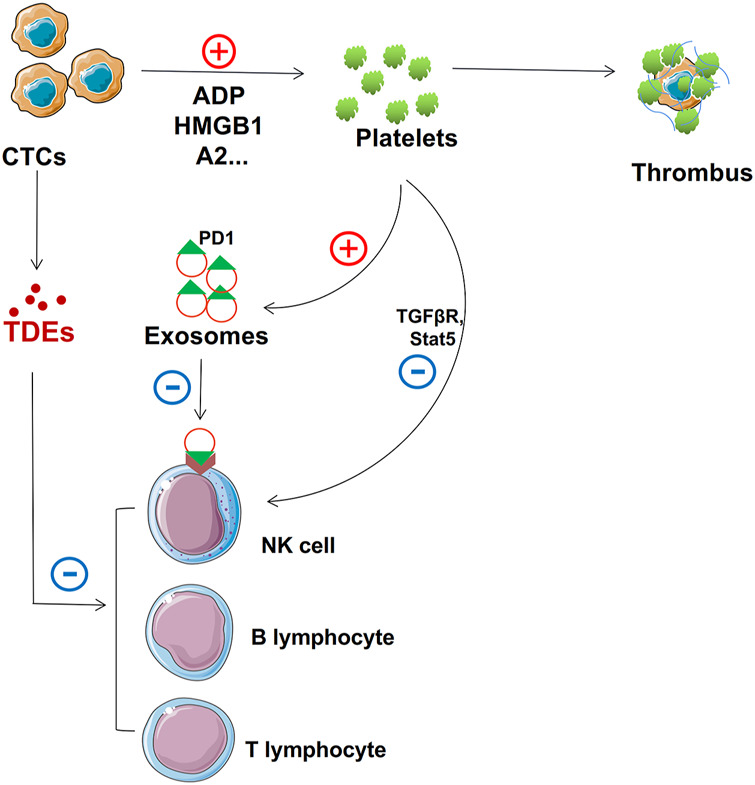
Role of TDEs in protecting CTCs. TDEs from CTCs and activated platelets suppress NK cell, T cell, and B cell function. In addition, activated platelets can form thrombi that adhere to the surface to protect CTCs.

Tumor cells shed from primary or secondary tumors are called circulating tumor cells (CTCs) (Paoletti and Hayes, 2016). CTCs invade the bloodstream and attach to the endothelium in the target organ. They then invade the surrounding parenchyma to form new tumors (Garcia et al., 2018). Blood is an unfavorable environment for CTCs, and they struggle with circulating immune cells (Agarwal et al., 2018). TDEs help CTCs metastasize smoothly by inhibiting immune cell activity and conferring a protective layer on them, thus maintaining cellular integrity (Figure 4).

### Tumor-Derived Exosomes can Suppress Immune Cells to Protect CTCs

The immune system inhibits the progression of cancer cells. Many immune cells are found circulating in human blood, including T lymphocytes, natural killer (NK) cells, and B lymphocytes (de la Cruz-Merino et al., 2008; Grivennikov et al., 2010; McCarthy, 2001). These immune cells play crucial roles in immune surveillance, immunosuppression, and killing effects and mainly act on CTCs (Deepak and Acharya, 2010; Pahl and Cerwenka, 2017; Wernersson and Pejler, 2014; Ye et al., 2017). Immune cells can recognize and attack CTCs under normal circumstances; therefore, immunosuppression is necessary for the metastasis of CTCs (Guo et al., 2019). Many researchers have found that TDEs can suppress immune cells. Exosomes carry bioactive molecules that can impair immune cell function (Becker et al., 2016; Kalluri, 2016; Robbins and Morelli, 2014). Programmed cell death receptor ligand 1 (PD-L1) can bind to programmed cell death protein 1 (PD-1) to inactivate T cells through its extracellular domain (L. Chen and Han, 2015; Chen et al., 2015; Garcia-Diaz et al., 2019). TDEs carry PD-L1 on their surface and suppress CD8^+^ T cell function in metastatic melanoma (Chen et al., 2018). In addition to PD-1, TDEs can also carry others to inhibit T cell function, and prolyl hydroxylase can inhibit CD4^+^ and CD8^+^ T cell functions by oxygen sensing (Clever et al., 2016). TDEs block T cell activation and enhance T cell apoptosis (Czernek and Dutchler, 2017; Ludwig et al., 2017). TDEs can also cause NK cell dysfunction. NK cells do not express PD-1; however, TDEs interfere with the TGFβ/TGFβRI/II pathway and other common molecular pathways, such as the adenosine pathway, eventually driving NK cell responses (Hong et al., 2017). In addition, TDEs can inhibit NK cell cytotoxicity by suppressing STAT5 activation (Zhang et al., 2007). B cells play a critical role in immunoglobulin, antigen, and proinflammatory cytokine secretion (Mauri and Bosma, 2012). TDE HMGB1 regulates the proliferation of T cell Ig and mucin domain-1^+^ (TIM-1^+^) B cells and fosters cancer cell immune evasion (Ye et al., 2018). We can design therapeutic modalities to enhance immune cell surveillance and killing of these tumors by understanding these signaling pathways.

### CTCs Activate Platelets Directly or by Releasing Exosomes

Platelets play major roles in hemostasis and coagulation and regulate the efficiency of canceration, tumor angiogenesis, tumor metastasis, and chemotherapy (Sharma et al., 2014). Platelets and cancer cells interact, thus affecting tumor growth and metastasis (Sharma P. et al., 2018). During blood circulation, other nontumor help is essential, for example, platelets can protect CTCs from blood flow shear forces by providing a protective layer. CTCs release soluble mediators, such as adenosine diphosphate (ADP), thromboxane (TX) A2, or high-mobility group box 1 (HMGB1), that can ligate toll-like receptor 4 (TLR4) to instigate localized platelet activation and form thrombus encasing tumor cells, thus protecting them from cytolysis by NK cells (Aitokallio-Tallberg et al., 1985; Nieswandt et al., 1999; Yu et al., 2014; Zucchella et al., 1989).

The interaction between platelets and CTCs can lead to platelet activation, and platelets release cytokines conducive to the survival and proliferation of tumor cells. When platelets combine with circulating tumor cells, platelet-derived soluble factors (TGF β and PDGF) mediate and prevent NK cells from detecting and dissolving tumor cells (Lambert et al., 2017; Lee J.-K. et al., 2013).

Finally, platelets prevent tumor cells from being eliminated by the immune system. Platelet-derived TGF-β can downregulate NKG2D expression and inactivate NK cells (Y. Chen et al., 2015; Kopp et al., 2009). The platelet expression profile in tumor and nontumor patients varies substantially (Santarpia et al., 2018). The interaction between CTCs and platelets can protect CTCs from immune surveillance during circulation and help tumor cells adhere to the endothelial cells at the metastasis site (Santarpia et al., 2018). Kuznetsov et al. showed that luminal breast cancer cells carried platelets that loaded factors with the effect of pro-inflammatory and pro-angiogenic activities and confirmed that these factors were released at distant tumors sites (Kuznetsov et al., 2012). Platelets are essential for releasing proangiogenic cytokines and recruiting angiogenic vascular endothelial growth factor receptor 2^+^ (VEGFR2^+^) cells that promote malignant progression (Schlesinger, 2018). Moreover, studies have shown that platelets may not just have a secondary role but may also drive malignant progression (or metastasis) (Kuo et al., 2011).

In human blood, platelets are considered to be the major contributors of exosomes (Caby et al., 2005). Goetzl et al. showed that endothelial cells absorb platelet-derived exosomes and enhance their adhesion by increasing endothelial cell adhesion protein expression and anti-adhesion factor production, thereby promoting CTC adhesion in vascular endothelial cells (Goetzl et al., 2016). Platelet-derived exosomes also increase platelet adhesion to monocytes and consequently monocyte activation, thus promoting the formation of inflammatory phenotypes (Goetzl et al., 2016).

Therefore, many researchers believe that blood platelets may be a potential source of biomarkers to aid cancer diagnosis. Nonetheless, the mechanism using which CTC-educated platelets mediate CTCs to avoid damage in the circulatory system still needs further research. We firmly believe that these CTC-educated platelet-derived exosomes play an important role in preventing damage to CTCs.

## Integrins of Tumor-Derived Exosomes Determine Organotropic Metastasis

That different types of cancer cells preferentially colonize and metastasize to different organs is the salient feature of metastasis (Nguyen et al., 2009). Current research shows that tumors mainly metastasize to lung, brain, lymph node, bone, and liver tissues. We have summarized organotropic metastasis with respect to cancer types (Table 1). Many studies focus on tumor cell adhesion function, and extracellular matrix molecules, such as integrins, have been determined to be related to the choice of organotropic metastasis (Valastyan and Weinberg, 2011).

**TABLE 1:** Chart for organotropic metastasis with respect to cancer types.

Cancer type	Organotropic metastasis	References
Acute myeloid leukemia	Liver metastasis	Wang H. et al. (2020); Wang N. et al. (2020)
Breast cancer	Bone metastasis	Salvador et al. (2019); Tahara et al. (2019); Ma et al. (2020a)
	Lung metastasis	Kim et al. (2018); Yousefi et al. (2018); Tyagi et al. (2021)
	Brain metastasis	Pedrosa et al. (2018); Chang et al. (2020); Hosonaga et al. (2020)
	Lymph node metastasis	Zhang H. et al. (2017); Qiu et al. (2020); Zhang Q. et al. (2017); Xu et al. (2021)
	Liver metastasis	Ma et al. (2015); Yousefi et al. (2018); Bale et al. (2019); Ji et al. (2020)
Bladder cancer	Bone metastasis	Fan et al. (2020)
	Lymph node metastasis	Doshi et al. (2013); Tuncer et al. (2014)
	Lung metastasis	
	Liver metastasis	
	Mediastinum	
	Adrenal gland	
Colon cancer	Liver metastasis	Yao et al. (2017); Tokoro et al. (2018); Kim et al. (2019a); Li K. et al., (2019); Zhu et al. (2020)
	Lung metastasis	Yao et al. (2017); Tokoro et al. (2018)
	Lymph node metastasis	(El-Halabi et al. (2014)
	Brain metastasis	Yoshida et al. (2012); Tokoro et al. (2018)
Cervical cancer	Lung metastasis	Ali et al. (2017); Chen et al. (2020); Hsieh et al. (2021)
	Brain metastasis	Hwang et al. (2013); Sato et al. (2015); Fetcko et al. (2017); Kim et al. (2019b); Sun et al. (2020)
	Lymph node metastasis	Wu et al. (2020); Zhang C. et al. (2020); Zhang Q. et al. (2020); Zhong et al. (2020)
	Bone metastasis	Yoon et al. (2013); Nartthanarung et al. (2014); Kanayama et al., (2015); Makino et al. (2016)
	Liver metastasis	Nance et al. (2020); Liu et al. (2021)
Gastrointestinal stromal tumor	Liver metastasis	Yamanaka et al. (2013); Yamashita et al. (2016); Cumsille et al. (2019)
	Bone metastasis	Aktan et al. (2015); Suzuki et al. (2015)
	Lymph node metastasis	Canda et al. (2008); Gong et al. (2011); Kubo and Takeuchi, (2017)
	Brain metastasis	Naoe et al. (2011)
	Lung metastasis	Xu et al. (2020a); Carvalho et al. (2020)
Gastric cancer	Lung metastasis	Qiu et al. (2018a); Wang et al. (2019d); Abe et al. (2020)
	Brain metastasis	York et al. (1999); Peng et al. (2014); Yang et al. (2016); Qiu et al., (2018b); Cavanna et al. (2018)
	Lymph node metastasis	Chen et al. (2019); Wang J. et al. (2020); Wang Y. et al. (2020); Kim et al. (2021)
	Bone metastasis	Ubukata et al. (2011); Mikami et al. (2017); Qiu et al. (2018a); Fujita et al. (2020); Imura et al. (2020)
	Liver metastasis	Zhang G. et al. (2017); Qiu et al. (2018b); Luo et al. (2019); Ohara et al. (2020)
Glioblastoma	Lung metastasis	Hoffman et al. (2017)
	Lymph node metastasis	Dolman, (1974); Jamjoom et al. (1997); Datta et al. (1998); Alhoulaiby et al. (2020)
	Bone metastasis	Ricard et al. (2019); Nagata et al. (2020)
	Liver metastasis	Yung et al. (1983); Shuto et al. (1995)
Hepatocellular carcinoma	Bone metastasis	Zhang et al. (2019b); Park et al. (2019); Ma et al. (2020b); Hu et al. (2020)
	Lymph node metastasis	Xu et al. (2016); Ikegami et al. (2017); Liu et al. (2018a)
	Stomach and colon	Kim et al. (2020)
	Brain metastasis	Yamakawa et al. (2015); Lin et al. (2017); Nam et al. (2019)
	Lung metastasis	Zhang et al. (2019a); Kapoor et al. (2020)
Head and neck cancer	Lung metastasis	Nishino et al. (1996); AlShammari et al. (2020)
	Brain metastasis	Nishino et al. (1996); Kofler et al. (2017)
	Lymph node metastasis	Zhou X. et al. (2018); Nienstedt et al. (2018); Zhou Z. et al. (2018); Mermod et al. (2019); Fang et al. (2020); Nishio et al. (2021)
	Bone metastasis	Bhandari and Jain, (2013); Chi et al. (2021)
	Liver metastasis	Chen et al. (2014)
Lung cancer	Liver metastasis	Sridhar et al. (2019); Wang B. et al. (2020); Lu et al. (2020)
	Bone metastasis	Liu et al. (2017); Okabe et al. (2018); da Silva et al. (2019); Wang et al. (2019b); Ai et al. (2020)
	Lymph node metastasis	Xia et al. (2015); Kong et al. (2017); Wang C.-F. et al. (2020); Wang L. et al. (2020)
	Brain metastasis	Aljohani et al. (2018); Waqar et al. (2018); Wang et al. (2019a); Luo et al. (2020b); Fujimoto et al. (2020)
Melanoma	Liver metastasis	Nakamura et al. (2017a); Ryu et al. (2017); Grossniklaus, (2019); Bustamante et al. (2021)
	Bone metastasis	Uluckan, (2019); Caldaria et al. (2020); Mannavola et al. (2020)
	Lymph node metastasis	Merkow et al. (2016); Moy et al. (2017); Muhsin-Sharafaldine et al. (2017); Faries et al. (2018); Soler-Cardona et al. (2018)
	Brain metastasis	Kircher et al. (2016); Katona et al. (2017); Redmer, (2018); Schwarz et al. (2019)
	Lung metastasis	Zhu et al. (2016); Hyun et al. (2020); Park et al. (2020); Stansel et al. (2020)
Multiple myeloma (BM-MSC)	Liver metastasis	Estelles Piera et al. (1993)
Mesothelioma	Liver metastasis	Etoh et al. (1997); Marzullo et al. (2020)
	Bone metastasis	Laurini, (1974); Swayne et al. (1992); Roegel et al. (1993); Huang et al. (2019)
	Lymph node metastasis	Sussman and Rosai, (1990); Yan et al. (2006); Abdel Rahman et al. (2008); Takehara et al. (2014)
	Brain metastasis	Asoh et al. (1990); Kawai et al. (1997); Hirooka et al. (2016)
Ovarian cancer	Lung metastasis	Ueda et al. (1973)
	Brain metastasis	Pakneshan et al. (2014); Stasenko et al. (2019)
	Lymph node metastasis	Kleppe et al. (2011); Zhou et al. (2016); Hong et al. (2018); Longo et al. (2020)
	Bone metastasis	Kumar et al. (1992); Tiwari et al. (2007); Zhang and Sun, (2013)
	Liver metastasis	Xu et al. (2017); Wang et al. (2019c); Zhuo et al. (2020)
Pancreatic cancer	Lung metastasis	Park et al. (2017); Sakimura et al. (2019); Uesato et al. (2020)
	Brain metastasis	Matsumura et al. (2009); Lemke et al. (2013); Matsumoto and Yoshida, (2015); Sasaki et al. (2019)
	Lymph node metastasis	Kanda et al. (2011); Liu et al., 2018b; Ma et al., 2018; Seifert et al. (2020)
	Bone metastasis	Saif et al. (2010); Guan et al. (2017); Outani et al. (2018)
	Liver metastasis	Ho et al. (2020); Wang et al. (2021a)
Prostate cancer	Lung metastasis	Polistina et al. (2020)
	Brain metastasis	Hernandez-Esquivel et al. (2018); Marchand Crety et al. (2020); Shida et al. (2020)
	Lymph node metastasis	Li F. et al. (2019); Xu et al. (2020b); Zhao et al. (2020); Klingenberg et al. (2021)
	Bone metastasis	Berish et al. (2018); Zhang, (2019); Wen et al. (2020)
	Liver metastasis	Simons et al. (2020); Ma B. et al. (2021)

Integrins, a large family of adhesion molecules, can mediate cell–cell and cell–extracellular matrix interactions (Desgrosellier and Cheresh, 2010). Many integrins are associated with tumor angiogenesis, such as αvβ3, αvβ5, and α5β1 (Cascone et al., 2005; Lee et al., 2013a; Huang and Rofstad, 2018). β1 integrins bind to vascular cell adhesion molecule 1 (VCAM-1) on ECs and play an important role in trans-endothelial migration (Klemke et al., 2007). Integrins participate in tumor angiogenesis by interacting with the VEGF–VEGFR and ANG–TIE pathways (Klemke et al., 2007). αvβ3 integrin binds to the adhesion molecule L1 on ECs driving trans-endothelial migration (Voura et al., 2001). αvβ3 integrin is the most abundant and influential receptor among integrins on ECs and can regulate angiogenesis (De et al., 2005; Mahabeleshwar et al., 2008; Shattil and Ginsberg, 1997). It can be activated and colocalized with VEGFR-2 on ECs of proliferating blood vessels (Mahabeleshwar et al., 2008). VEGF-stimulated c-Src can be the phosphorylate β3 subunit on ECs, promoting VEGFR-2 phosphorylation and activation (De et al., 2005; Mahabeleshwar et al., 2008; Mahabeleshwar et al., 2007). In addition, αvβ3 is necessary for the survival and maturation of new blood vessels, and proliferative angiogenic EC apoptosis occurs after treatment with αvβ3 antagonists (Brooks et al., 1994). Briefly, integrin subunits α1, α2, α3, α4, α5, α6, α9, αv, β1, β3, and β5 are involved in physiological or pathological angiogenesis. Exosomes affect several steps of angiogenesis including motility, cytokine production, cell adhesion, and cell signaling (Taverna et al., 2012). These can improve the tumor survival environment before metastasis.

**FIGURE 5:**
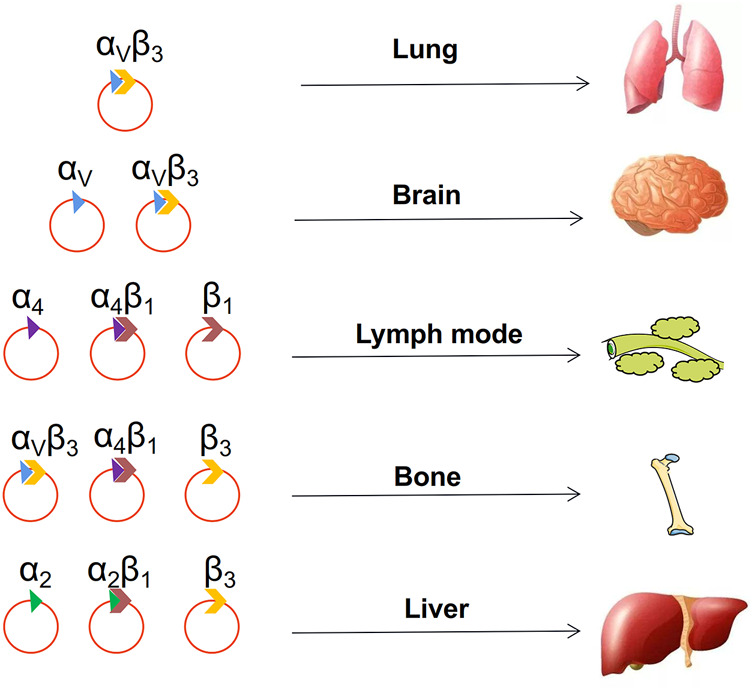
Choice of metastatic organs. The integrin on the surface of the exosome determines where the tumor metastasizes.

Although integrins are secreted by tumor cells, it is transported by exosomes to a distant organ (Peinado et al., 2017). Lyden et al. showed that tumor exosome integrins can determine organotropic metastasis. They suggested that tumor exosome integrins can fuse with organ-specific resident cells and activate Src phosphorylation and proinflammatory S100 expression to establish a pre-metastatic niche (Hoshino et al., 2015). In addition, more bodies of evidence identified that different integrins on the surface of exosomes play varied roles in metastasis to specific organs (Alderton, 2015; Hoshino et al., 2015; Paolillo and Schinelli, 2017). For instance, exosomal integrins α6β4 and α6β1 preferentially direct tumor cells to the lungs, and αvβ5 induces liver metastasis (Hoshino et al., 2015). Tumor exosomes can prepare pre-metastatic niches to facilitate organ-specific metastasis, even for cancer cells equipped to metastasize (Figure 5).

## Tumor Cell Growth at the Metastasis Site

Once tumor cells migrate to tissues and organs, TDEs provide them with a good growth environment and the ability to promote their growth.

### Tumor-Derived Exosomes Promote Pre-metastatic Niche Formation

A pre-metastasis niche is a primary tumor in secondary organs and tissues that creates a favorable microenvironment for subsequent metastasis. Tumor-derived molecules secreted by primary tumors play a key role in preparing distant sites for the formation of new pre-metastasis niches, promoting metastasis and even determining the orientation of metastatic organs. These major tumor-derived molecules are usually tumor-derived secretory factors, extracellular vesicles (EVs), and other molecular components (Minciacchi et al., 2015). Exosomes containing protein, mRNA, or DNA fragments promote the pre-metastasis niche formation by mediating the communication between tumor cells and surrounding components or transferring their contents to recipient cells (Chin and Wang, 2016; Zhou et al., 2014).

Tumor cells are “seeds”. With tumor-secreting factors, tumor cell–secreting vesicles, and exosomes acting as catalysts, tumor cells can promote the formation of the “soil” (host microenvironment) in a distant metastasis site and promote the growth of cancer cells. Cancer metastasis is preceded by the interaction between the seed and soil (Y. Chen et al., 2015; Lambert et al., 2017; Liu and Cao, 2016; Minciacchi et al., 2015). Primary tumor cells influence and change the microenvironment at secondary organs by promoting pre-metastasis niche factor before tumor cells arrive (Chin and Wang, 2016; He et al., 2017).

The characteristics of a pre-metastasis niche include the following six aspects. First, pre-metastasis niche formation is accompanied by the recruitment of bone marrow–derived cells (BMDC) (Y. Chen et al., 2015; Minciacchi et al., 2015). Literature suggests that extracellular matrix metalloproteinase inducer (EMMPRIN) in cancer cells can induce the secretion and expression of many factors, such as SDF and VEGF, which mediate the recruitment of BMDC to the liver and lungs (Y. Chen et al., 2015; Minciacchi et al., 2015). Second, the immune cells involved in the pre-transfer niche formation are heterogenous. Pre-metastasis niche formation involves not only the recruitment of foreign cells but also the reprogramming of resident stromal cells, promoting metastasis. Pre-metastasis niche formation also involves the change of the ECM. Niche formation before transfer is accompanied by a change in the vascular system. Metastatic breast cancer cells reduce tight junctions between endothelial cells by secreting exosomes containing mir-105, thus inducing systemic vascular leakage and promoting metastasis (Kong et al., 2019). Breast cancer cells secrete exosomes containing miR-122, which are absorbed by niche cells, and reduce glucose consumption by targeting pyruvate kinase, thus increasing the proliferation rate and survival rate of cancer cells and promoting metastasis (Fong et al., 2015). Pancreatic cancer–derived exosomes, rich in macrophage migration inhibitory factors, recruit macrophages and induce pre-metastasis niche formation in the liver (Costa-Silva et al., 2015). Modulation of the pre-metastatic niche formation by controlling TDEs is a new area for future chemotherapy research.

### Tumor-Derived Exosomes Promote the Growth of Metastasis Tumor

**FIGURE 6:**
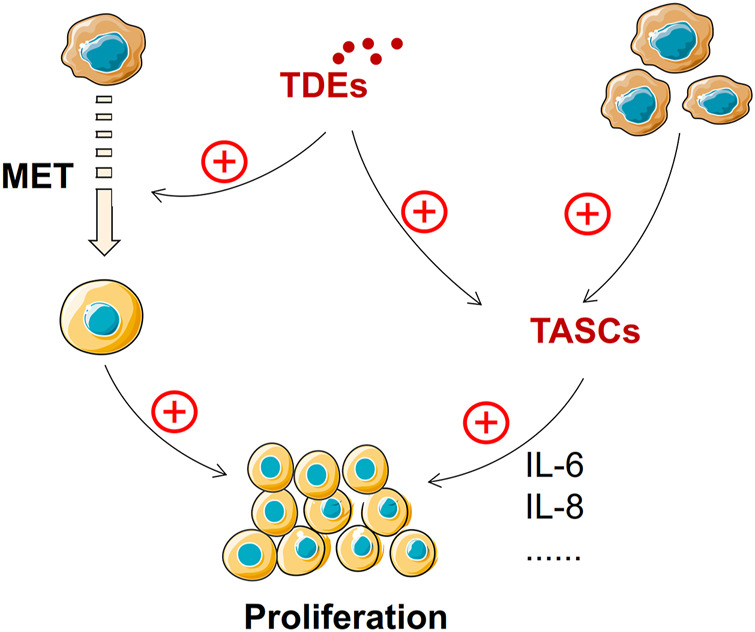
TDEs promote tumor growth at the metastasis site. MET and TASCs promote tumor growth at metastatic sites, and TDEs can derive MET and TASC formation.

The growth of metastatic tumors requires suitable “soil”. MET returns the cancer cells to a highly proliferative state but with the loss of their migration characteristics, enabling tumor growth at the metastasis site (Li K. et al., 2019). The characteristics of MET are increased expression of mesenchymal markers, such as vimentin, and decreased expression of epithelial markers, such as E-cadherin, compared with that of EMT (Wells et al., 2008). MET supports the reacquisition of epithelial features to promote metastasis (Brabletz et al., 2001). Several signaling pathways are involved in regulating MET, including transforming growth factor (TGF), fibroblast growth factors (FGFs), bone morphogenic protein (BMP), epidermal growth factor receptor (EGFR), hepatocyte growth factor (HGF), Wnt/β-catenin, and Notch pathways (Said and Williams, 2011). TDEs can support tumor progression and remodel surrounding parenchymal tissues at the metastatic site (Greening et al., 2015). TDEs play an important regulatory role mediating EMT and transforming MET (Bigagli et al., 2019). Gastric cancer cell–derived exosomes can mediate the stimulation of proinflammatory cytokine IL-1β secretion and activate the Akt and MAPK pathways to promote tumor growth at the metastatic site (Che et al., 2018; Wang et al., 2019e). In addition, TDEs can transform stromal cells into tumor-associated stromal cells (TASCs) that can secrete many pro-tumorigenic factors, including IL-6 and IL-8. These factors can enhance the proliferation ability of tumor cells (Bussard et al., 2016). Hence, TDEs can enable tumor cells to acquire proliferation capacity directly through the MET process or promote tumor proliferation by inducing TASC formation and releasing related factors (Figure 6). Nevertheless, there is still a dearth of research on exosomes and their contribution to MET.

## Conclusion

Exosomes play an important role in every step leading to tumor metastasis. Although there are many reports on the role of exosomes in metastasis, much is left to be explored of the potential mechanisms underlying metastasis. Although a few studies still have unclear results, we have summarized the published literature on the substances that exosomes carry, their main functions in different tumors, the target cells affected, and steps involved in metastasis. (Table 2). Exploring these underlying mechanisms will enlighten us about cancer biology and contribute to the prevention of and therapeutic strategies for malignancies. We can manipulate TDEs to impede not just metastasis formation but even established metastases.

**TABLE 2:** Role and target of the components of TDEs in tumor metastasis.

Cancer type	Exosome component	Target cells	Potential regulation	Roles in metastasis steps	References
AML	TGF-β	NK cells	NKG2D	Step 3: immunosuppressive	Szczepanski et al. (2011)
	DPP4	Bone	-	Step 5	Namburi et al. (2021)
	TGFβ/TGFβRI/II	NK-92	-	Step 3: immunosuppressive	Hong et al. (2017)
Breast cancer	miR-10b	Mammary epithelial cells	HOXD10 and KLF4	Step 1: enhance invasion ability	Singh et al. (2014)
	miR-122	Lung fibroblast neurons	PKM	Step 2: non-coding RNA influence angiogenesis	Fong et al. (2015)
	RN7SL1	Breast cancer cells	PRR RIG-I	Step 5	Nabet et al. (2017)
	miR-200c, miR-141	Breast cancer cells	FOXP3-KAT2B	Enhance metastases	Zhang G. et al. (2017)
	miRNA-503	Microglia	-	Step 3: immunosuppressive	Xing et al. (2018)
	Caveolin-1	Breast cancer cells	-	Enhance metastases	Campos et al. (2018)
	miR-193b	Breast cancer cells	RAB22A	Step 1: enhance invasion ability	Sun et al. (2018)
	CEMIP	Brain endothelial and microglial cells	-	Step 2: angiogenesis	Rodrigues et al. (2019)
	hsa-miR-940	Osteoblastic	ARHGAP1 and FAM134A	Step 5	Hashimoto et al. (2018)
	miR-126a	Lung	S100A8/A9	Step 2	Deng et al. (2017)
	miR-222	Breast cancer cells	NF-κB	Step 1	Ding et al. (2018)
	miR-130a-3p	Breast cancer cells	RAB5B	Step 1	Kong et al. (2018)
	miR-939	Breast cancer cells	VE-cadherin	Step 2: non-coding RNA influence angiogenesis	Di Modica et al. (2017)
	miR-770	TNBCs	STMN1	Decrease metastases	Li Y. et al. (2018)
	miR-4443	Breast cancer cells	TIMP2	Step 4	Wang H. et al. (2020)
	miR-210	Endothelial cells	-	Step 2: non-coding RNA influence angiogenesis	Kosaka et al. (2013)
	miR-1910-3p	Breast cancer cells	MTMR3	Step 1: enhance invasion ability	Wang B. et al. (2020)
	miR-146a	CAFs	TXNIP	Step 5	Yang et al. (2020b)
	miR-4443	Liver	-	Step 1: enhances invasion ability	Wang J. et al. (2020)
Bladder Cancer	LINC02470, LINC00960	Bladder cancer cells	-	Step 1: EMT	Huang et al. (2020b)
CML	miR-92a	EC	Integrin α5	Step 2: non-coding RNA influence angiogenesis	Umezu et al. (2014)
Colon cancer	hsp 70	MDSC	STAT3	Step 3: immunosuppressive	Chalmin et al. (2010)
	KRAS mutation	Colon CA cells	-	Step 5: tumor growth	Demory Beckler et al. (2013)
	TF	EC	-	Step 3: platelet activation	Garnier et al. (2012)
	miR-193a	Colon cancer cells	Caprin1	Step 5: decrease the growth of cells	Teng et al. (2017)
	miR-92a-3p	Colon cancer cells	-	Step 1: EMT	Hu J. L. et al. (2019)
	lncRNA H19	Colon cancer cells	miR-141	Step 5: MET	Ren et al. (2018)
	miR-21-5p; miR-155-5p	Colon cancer cells	BRG1	Step 5	Lan et al. (2019)
	miR-182-3p	Colon cancer cells	FOXO4	Step 1: EMT	Liu et al. (2019)
	GDF15	HUVECs	Smad	Step 2	Zheng et al. (2020)
	MCP-1; TNF	Macrophages	-	Step 2: activating macrophages	Chen et al. (2016)
	miR-25-3p	ECs	VEGFR, ZO-1, occludin, and claudin5	Step 2: angiogenesis	Zeng et al. (2018)
	miR-1229	ECs	HIPK2	Step 2: angiogenesis	Hu H.-Y. et al. (2019)
Cervical cancer	Survivin	Cervical cancer cells	-	Step 5: tumor growth	(Khan et al., 2009; Khan et al., 2011)
	Cicr-PVT1	Cervical cancer cells	MiR-1286	Step 1: EMT	Wang H. et al. (2020)
	miR-221-3p	HLEC	VASH1	Step 2: Lymphatic vessel formation	Zhou et al. (2019)
	miR-663b	Cervical cancer cells	MGAT3	Step 1: EMT	You et al. (2021b)
GIST	KIT	Progenitor muscle cells	MMP1	Step 1: Influence the relationship between tumor cells and cell matrix	You et al. (2021b)
Gastric cancer	miR-27a	CAFs	-	Step 1: EMT	Wang J. et al. (2018)
	miR-130a	ECs	C-MYB	Step 2: angiogenesis	Yang et al. (2018)
	miR-135b	ECs	FOX1	Step 2: angiogenesis	Bai et al. (2019)
Glioblastoma	EGFR vIII	Glioblastoma cells	VEGF, Bcl-x (L), p27	Step 5: tumor growth	Al-Nedawi et al. (2008)
	matrix metalloproteinases, IL-8, PDGFs, and caveolin 1	Glioblastoma cells	PI3K/AKT	Step 1: EMT	Kucharzewska et al. (2013)
	L1CAM	Glioblastoma cells	FAK; FGFR	Enhance metastases	Pace et al. (2019)
	miR-148a	Glioblastoma cells	CADM1	Step 1	Cai et al. (2018)
	MDA-9/Syntenin	Glioblastoma cells	CD63-AP-2	Step 1	Das et al. (2018)
	LncRNA CCAT2	ECs	-	Step 2: angiogenesis	Lang et al. (2017a)
	LncRNA POU3F3	ECs	-	Step 2: angiogenesis	Lang et al. (2017b)
HCC	miR-584, 517c, 378	HCC cells	TAK1	Step 5: tumor growth	Kogure et al. (2011)
	miR-1247-3p	Fibroblasts	B4GALT3	Step 5: TASCs	Fang T. et al. (2018)
	miR-122	HCC cells		Step 5: tumor growth	Qian and Pollard, (2010)
	miR-27b-3p/miR-92a-3p	HCC cells	IGF1R	Step 5: tumor growth	Basu and Bhattacharyya et al. (2014)
	miR-103	ECs	VE-cadherin	Step 2: non-coding RNA influence angiogenesis	Basu and Bhattacharyya et al. (2014), Fang J. H. et al. (2018)
	miR-21, miR-10b	HCC cells	-	Step 1	Tian et al. (2019)
	SMAD3	HCC cells	ROS	Step 4: attach	Fu et al. (2018)
				Step 5: tumor growth	
	LOXL4	HUVECs	FAK/Src	Step 2: angiogenesis	Li R. et al. (2019)
	Vps4A	HCC cells	β-catenin	Step 1: EMT	Han et al. (2019)
	miR-320a	HCC cells	CDK2, MMP2	Step 1: EMT	Zhang Z. et al. (2017)
				Step 5: TASCs	
	lncRNA FAL1	HCC cells	miR-1236	Enhance metastases	Li B. et al. (2018)
	p120-catenin	HCC cells	STAT3	Enhance metastases	Cheng et al. (2019)
	miR-372-3p	HCC cells	Rab11a	Enhance metastases	Cao et al. (2019)
	Alpha-enolase	HCC cells	Integrin α6β4	Enhance metastases	Jiang et al. (2020)
	circ_MMP2	HCC cells	MMP2	Enhance metastases	Liu et al. (2020)
	miR-92a-3p	HCC cells	PTEN/Akt	Step 1: EMT	Liu et al. (2020)
	Linc00161	HUVECs	miR-590-3p/ROCK	Step 2: angiogenesis	You et al. (2021a)
	miR-30a; miR-222	HCC cells	MIA3	Enhance metastases	Du et al. (2021)
	S100A4	HCC cells	STAT3	Enhance metastases	Sun et al. (2021)
	miR-1290	ECs	SMEK1	Step 2: angiogenesis	Wang et al. (2021b)
	circRNA-100338	HUVECs	-	Step 2: angiogenesis	Huang et al. (2020b)
	TIM11	B cells	TLR/MAPK	Step 3: immunosuppressive	Ye et al. (2018)
HNC	FasL	T cells	Jurkat	Step 3: immunosuppressive	Kim et al. (2005)
	miR-23a	HUVECs	TSGA10	Step 2: angiogenesis	Bao et al. (2018)
	-	NK cells	NKG2D	Step 3: immunosuppressive	Ludwig et al. (2017)
Lung Cancer	miR-103	M2 macrophages	VEGF-A	Step 2: angiogenesis	(Hsu et al., 2018; Wu et al., 2019)
	miR-23a	ECs	ZO-1	Step 2: angiogenesis	Hsu et al. (2017)
	miR-21	HUVECs	-	Step 2: angiogenesis	Liu et al. (2016a)
	LncRNA-p21	HUVECs	-	Step 2: angiogenesis	Castellano et al. (2020)
Melanoma	MET	BM progenitor cells	-	Step 5: tumor growth	Peinado et al. (2012)
	PD-L1	T cells	PD-1	Step 3: immunosuppressive	Chen et al. (2018)
	snRNA	Lung epithelial cells	TLR3	Step 5: TASCs	Liu et al. (2016b)
	CD151	Lung, lymph node and stromal cells	-	Step 4: location	Malla et al. (2018)
	Fas	T cells	MMP9	Step 3: immunosuppressive	Cai et al. (2012)
	miR-191; let-7a	Melanoma cells	-	Step 1: EMT	Xiao et al. (2016)
	Immunomodulatory, proangiogenic factors	Melanoma cells	-	Step 2: angiogenesis	Ekstrom et al. (2014)
				Step 3: immunosuppressive	
	HSP70	NK cells	-	Step 3: immunosuppressive	Elsner et al. (2007)
	uPAR	HMVECs; ECFCs	ERK1,2	Step 2: angiogenesis	Biagioni et al. (2021)
	miR-106b-5p	Melanoma cells	EphA4	Step 5: MET	Luan et al. (2021)
	miR-155-5p	CAFs	SOCS1/JAK2/STAT3	Step 2: angiogenesis	Zhou X. et al. (2018)
Multiple myeloma (BM-MSC)	miR-15a	MM cells	FAK	Step 1: enhance invasion ability	Roccaro et al. (2013)
	miR-let-7c	ECs	-	Step 2: TDEs promote angiogenesis by activating macrophages	Tian et al. (2021)
	miR-135b	EC	HIF-FIH	Step 2	Umezu et al. (2014)
Mesothelioma	TGF-β	Fibroblasts	SMAD	Step 1: influence the relationship between tumor cells and cell matrix	Webber et al. (2010)
NPC	HIF1α	NPC cells	LMP1	Step 1	Aga et al. (2014)
	miR-23a	EC	TSGA10	Step 2: angiogenesis	Bao et al. (2018)
	MMP13	NPC cells	-	Step 1	You et al. (2015)
				Step 2: angiogenesis	
	circMYC	NPC cells	-	Enhance metastases	Luo et al. (2020a)
	LMP1	NPC cells	-	Step 1: EMT	Aga et al. (2014)
Ovarian cancer	FasL	T cells	CD3-zeta	Step 3: immunosuppressive	Taylor et al. (2003)
	ATF2; MTA1; ROCK1/2	HUVECs	-	Step 2: angiogenesis	Yi et al. (2015)
	GNA12; EPHA2; COIA1	MSCs; ECs	-	Step 5	Sharma et al. (2018b)
	CD44	HPMCs	-	Step 1	Nakamura et al. (2017a)
	circWHSC1	HPMCs	miR-145; miR-1182	Step 2	Nakamura et al. (2017b); Zong et al. (2019)
	miR-375	Ovarian cancer cells	CA-125	Enhance metastases	Su et al. (2019)
	miR-7	EOC	EGFR, AKT, ERK1/2	Decrease metastases	Hu et al. (2017)
	LncRNA FAL1	Ovarian cancer cells	PTEN/AKT	Enhance metastases	Hu et al. (2017)
	miR-6780b-5p	Ovarian cancer cells	-	Step 1: EMT	Cai et al. (2021)
	circRNA051239	Ovarian cancer cells	-	Step 5	Ma R. et al. (2021)
	LncRNA MALAT1	HUVECs	-	Step 2: angiogenesis	Qiu et al. (2018a)
Pancreatic cancer	MIF	Liver Kupfer cells	-	Step 5: tumor growth	Costa-Silva et al. (2015)
	miR-301a-3p	Macrophages	PTEN/PI3Kγ	Step 2: active macrophages	Wang X. et al. (2018)
	circ-IARS	HUVECs	-	Enhance metastases	Li J. et al. (2018)
	Lin28B	CAFs	let-7, HMGA2, PDGFB	Step 5	Zhang et al. (2019c)
	miR-501-3p	Pancreatic ductal adenocarcinoma	TGFBR3, TGF-β	Enhance metastases	Yin et al. (2019)
	lncRNA Sox2ot	Pancreatic ductal adenocarcinoma	-	Step 1: EMT	Li Z. et al. (2018)
	CD151, Tspan8	ASML	-	Step 1: matrix degradation	Yue et al. (2015)
	miR92a-3p	Pancreatic ductal adenocarcinoma	PTEN/Akt	Step 1: EMT	Yang et al. (2020a)
	CD44v6/C1QBP	Pancreatic ductal adenocarcinoma	-	Step 5	Xie et al. (2021)
Prostate cancer	αvβ6 Integrin	Prostate cancer cells	-	Step 4	Lazaro-Ibanez et al. (2014)
	miR-1246	Prostate cancer cells	N-cadherin; vimentin	Step 1: EMT	Bhagirath et al. (2018)
	miR-940	Osteoblastic	ARHGAP1, FAM134A	Enhance metastases	Bhagirath et al. (2018)
	miR-26a	Prostate cancer cells	-	Step 1: EMT	Wang et al. (2019f)
	PKM2	BMSCs	CXCL12	Step 5	Dai et al. (2019)
	PSGR	Prostate cancer cells	-	Step 1: EMT	Li et al. (2021)
	TGF-β2, TNF1α, IL6, TSG101, Akt, ILK, β-catenin	Prostate cancer cells	-	Step 1: matrix degradation	Ramteke et al. (2015)

The role of exosomes in various cancer metastases. AML: acute myeloid leukemia. CML: chronic myeloid leukemia. GIST: gastrointestinal stromal tumor. HCC: hepatocellular carcinoma. HNC: head and neck cancer. NPC: nasopharyngeal carcinoma.
